# Step-up, step-down mental health care service: evidence from Western Australia’s first – a mixed-method cohort study

**DOI:** 10.1186/s12888-020-02609-w

**Published:** 2020-05-11

**Authors:** Hanh Ngo, Priscilla Ennals, Serhat Turut, Elizabeth Geelhoed, Antonio Celenza, Keren Wolstencroft

**Affiliations:** 1 Division of Emergency Medicine, School of Medicine, The University of Western Australia (Internal Mail Code M516), 35 Stirling Highway, Crawley, WA 6009 Australia; 2 Neami National, Melbourne, VIC Australia; 3 School of Allied Health, The University of Western Australia, Perth, WA Australia; 4 Neami National, Wollongong, NSW Australia

**Keywords:** Community based mental health service, Sub-acute, Step-up step-down, Recovery, Prevention, Outcome measures, Patient satisfaction, Evaluation

## Abstract

**Background:**

Mental health Step-up, Step-down services (SUSD), also known as subacute services or Prevention and Recovery Services, have emerged to fill an identified gap between hospital-based inpatient care and clinical community-based mental health support. Evidence for the effectiveness of the SUSD service model is limited but growing. Accordingly, this study looked to add to the extant body of knowledge, by (i) assessing change outcomes in mental health and wellbeing, and predictors of these changes, for patients who accessed Western Australia’s first SUSD service; and (ii) evaluating patients’ satisfaction with service, and what patients value from their stay.

**Methods:**

This was a mixed-method retrospective cohort study. Participants comprised 382 patients who accessed a 22-bed Mental Health SUSD facility and incurred 551 episodes of care during the 01/07/2014–30/06/2016 period. Patients’ change outcomes in psychological distress, general self-efficacy, and work and social adjustment from service entry to service exit were analyzed using generalized linear modeling. Simple Pearson’s correlation coefficients were calculated for preliminary assessment of the associations between patients’ service satisfaction and their change outcomes. Qualitative outcomes that patients valued from their stay were analyzed thematically according to a semi-grounded theoretical approach.

**Results:**

Significant improvements were observed in patients’ self-reported psychological distress, self-efficacy, and work and social adjustment (all *p* < 0.0001). A strong and persistent baseline effect existed across the three measures. Older age, female gender, and having a dependent child in the same household were protective/enhancing factors for the patients’ recovery. Satisfaction with service was high. Patients valued having the time and space to recuperate, gain insight, focus, and create changes in their lives.

**Conclusion:**

The encouraging findings, regarding both patients’ change outcomes and satisfaction with service, support the value of the SUSD service model for patients with mental illnesses. Strengths and limitations were discussed; ensued recommendations were offered to both service providers and researchers to enhance the robustness of future research findings, to help inform more effective policy and funding decisions related to mental health care.

## Background

Mental health policy in Australia is undergoing reform to deliver a stepped care approach, to ultimately see people receiving supports matched to their level of need; that is, the right level of the right type of clinical care is received at the right time [1]. This requires access to a full continuum of services from low intensity, early intervention services through to acute inpatient services. As part of this reform process mental health Step-up, Step-down services (SUSD), also known as subacute services or Prevention and Recovery Services (PARCS), have emerged to fill an identified gap between hospital based inpatient care and clinical community based mental health support. Adult SUSD mental health services provide short- to medium-term residential care, either as a means of early intervention (Step-up) or to support the transition from hospital to home (Step-down). Since 2003, SUSDs have been embedded in the Victorian mental health service system; other Australian states have also begun to adopt them [2].

The Australian literature on the SUSD model is currently limited but growing. Existing studies have described aspects of the model as applied in different service settings such as location, capacity, goals and parameters, service usage demographics and service approach and program activities [3–9]. In addition, most of these studies include an evaluative component such as stakeholder perspectives in relation to how practice aligns with the service approach and patients’ experiences of service.

In the historic and international context, the SUSD model can be said to have evolved from the movement of ‘deinstutionalization’ beginning several decades ago in countries such as the United Kingdom (UK) and the United States (US) [10]. In the recent years, despite its uptake in several other countries worldwide, Australia included, the understanding of the SUSD model, or of stepped care in general, in terms of utility, scope, effectiveness and cost, remains limited [11]. This is because of the widely varied nature of the specific models implemented in each country, or each setting. Such parameters may include: admission/inclusion criteria, diagnosis, typical length of stay offered, types of service (e.g., clinical, educational, psychosocial etc) and service provider (e.g., government, private, not-for-profit etc) [12, 13]. For example, publications [14, 15] from a study of 17 SUSD services in one Australian state highlighted that while care was generally recovery-oriented there was wide variation in the “structure, resourcing, and content and quality of care” ([15],p.10). This study reinforced how local service systems, beyond the SUSD, influence the operation of SUSD services. With these varied characteristics, it is expected that different service models look to achieve different ‘outcomes’ [13]. In addition, SUSD-type services have been developed to address the immediate needs of certain target patient populations and local regions, with less focus on the additional tasks of evaluation and research [16].

At the more fundamental level, outcomes for the service users, referred to as patients henceforth, which purportedly are a key driver for the birth of this type of models, have been variably assessed using different measures, with mixed rationales. Examples of the measures used in relevant studies thus far include: the Mental Health Recovery Star (MHRS [4];), Health of the Nation Outcome Scales (HoNOS [4, 8] [9];);, Kessler Psychological Distress Scale (K10 [4];), Behaviour and Symptom Identification Scale (BASIS-32 [8] [9];);, Life Skills Profile – 16 (LSP-16 [9];), Scottish Recovery Indicator (SRI 2 [3];), Individual Recovery Plans (IRP’s [3];), and Assessment of Quality of Life (AQoL [17];). Findings from these studies provide an early indication that the SUSD has value for people beyond reductions in hospital usage, but exactly what the additional values are remain to be defined.

This lack of clarity in choice and rationale of outcome measures used in evaluating the SUSD service model is understandable, given the relatively new and unique position of this type of model, compared with the more traditional, or more established care models in the acute hospital setting, or the day-visit community setting [18, 19]. That is, the needs of the patients accessing SUSD services may differ from those in the other care settings. As also seen in the above summary of previous research, this diversity, and complexity, in choice and rationale of outcome measures, becomes even more compounded as one also considers whether the recovery/change outcomes for the patients themselves are also congruent with the outcomes expected of, and/or by, the service providers and funders. More specifically, in Australia, government funding goals for SUSDs are predominantly framed in relation to system and financial efficiencies, improvements in service delivery and better health outcomes [20].

There is also a case for pitching service outcome goals and measurement instrument design to ‘what is wanted’ in terms of quality of life and wellbeing outcomes that go beyond the absence of illness [21–25]. Exploring outcomes from the patients’ perspective in terms of the value and contribution of a stay in the SUSD to their overall lives may add to the knowledge in this area [25–29].

Taken together, while there is a small and growing volume of early research suggesting the value of the SUSD model, it is clear that substantial further research is needed to shed more light on the diverse facets of its effectiveness. Meaningful assessment of the model’s effectiveness can be realized via the adoption and/or development of measures of change or recovery that are relevant or salient to the patients, as well as informative to both the service providers and the health system collectively. In this light, the current study is set out to primarily assess change outcomes in mental health and wellbeing for a cohort of patients who accessed Western Australia’s Joondalup SUSD service, which is the first of its kind in this Australian state. At set-up, the service administrators conceptualized the Joondalup SUSD as being a setting where people make recovery gains that are assisted by the development of skills and confidence to self-manage upon return to home.

Recovery in this SUSD context is defined by “a deeply personal, unique process of changing one’s attitudes, values, feelings, goals, skills and/or roles” [30], and “the establishment of a fulfilling, meaningful life and a positive sense of identity founded on hopefulness and self-determination” [31]. Accordingly, to assess change outcomes, two measures recommended use in Australian settings – the Kessler 10 (K10 [32];), to measure level of psychological distress, and the Work and Social Adjustment Scale (WSAS [33];) – along with an additional measure, the General Self-Efficacy Scale (GSES [34];), were chosen as aligning with the service goals. The study also evaluates the patients’ experience with the service, namely their service satisfaction, and what they value from their service stay. The purpose of this secondary component is to seek to validate the relevance of the three mentioned scales as measures of the patients’ change outcomes in a SUSD setting.

## Methods

### Design

This was a retrospective cohort study, with mixed methods for data collection and analysis. The main part of the study, on patients’ change outcomes, utilized a simple pre-post design (with data collected at both service entry and exit) and quantitative data analysis. The secondary component of the study has a cross-sectional design, where data on patients’ satisfaction with, and perception of, the service, were collected only at service exit. This secondary component involved both quantitative and qualitative data analyses.

### Settings

The 22-bed facility, Neami Joondalup Mental Health Step-Up Step-Down service (JMHSS), is run by Neami National, a not-for-profit community mental health service supporting people with mental illness “to improve their wellbeing, live independently and pursue a fulfilling life” [35]. Neami as a psychosocial provider is directly contracted by WA Mental Health Commission to deliver the service. Neami employs mixture of psychosocial and clinical staff, for example registered nurses, enrolled nurses and allied health clinicians. A memorandum of understanding underpins the relationship with the local clinical mental services – inpatient and community based [36]. In addition to meeting the needs of the local region it is a statewide service accepting patients from across much of Western Australia. In 2013, JMHSS received its first patients. The JMHSS’s service practice is underpinned by a recovery-oriented strengths-based coaching framework – The Collaborative Recovery Model [37]. The framework is designed to support personal empowerment and self-agency through the development of individualized wellbeing plans based on personal aspirations and context [37–39]. Program activities such as The Optimal Health Program [40] are delivered to enhance self-efficacy and skills in wellbeing planning and management.

### Participants and data source

Participants were patients with mental health issues who accessed JMHSS service any time during the period from 1 July 2014 to 30 June 2016 inclusive. Patients’ data were routinely collected by the service provider JMHSS (part of Neami National) at service entry and exit. Patients may choose, or refuse, to provide the data in question. Patients are aware that Neami National may use the data for research and/or service evaluation and improvement purposes, provided that the data are anonymized in reporting and dissemination. In the specific context of this study, relevant patients’ data were made available in de-identified format to the first author, an independent researcher external to Neami National, for analysis.

### Materials

Patients accessing the JMHSS service were requested, but not obligated, to complete self-report measures of their state of health and functioning (namely: K-10, GSES, and WSAS, as mentioned above), in addition to providing their contact and demographic details. The K-10 consists of 10 questions, with each rated from 1 to 5 in order of increasing frequency of a particular (negative) feeling; thus a person may score as low as 10 (i.e., least distress) or as high as 50 (most distress). The GSES also has 10 items, with each rated from 1 to 4; hence total scores may range from 10 to 40, with higher scores indicating high self-efficacy. The WSAS has 5 items, with each rated from 0 (no impairment at all) to 8 (severe impairment), yielding a maximal possible range of 0 to 40 for total scores. All three scales K-10, GSES, and WSAS have been validated widely in other mental health service settings ([32–34]). In this study, these instruments were completed by the patients at treatment entry and exit. Patients were also invited to complete an Exit Questionnaire on their exit, to provide feedback on their experience of their stay at JMHSS (see Additional file 1).

### Outcome measures and definitions

Outcome measures were score changes in K-10, GSES, and WSAS – from service entry to service exit, and patients’ satisfaction with service upon exit. Score changes were defined as “score at exit minus score at entry”. It is noted that scales K-10 and WSAS measure ‘undesired’ constructs, namely psychological distress and work and social impairments. As such, a negative score change on K-10 or WSAS (i.e., a reduction in distress or impairment) is desired. The GSES, on the other hand, measures self-efficacy, a ‘desired’ construct. Therefore, a positive score change on GSES (i.e., an increase in self-efficacy) is desired.

### Statistical analysis

First, the study cohort was described using descriptive statistics: mean and standard deviation for continuous measures, and frequency and percentage for categorical measures. Descriptions involved demographic, clinical, and systemic characteristics. Demographic characteristics included age, gender, socio-economic indexes for areas (SEIFA [41];), country of birth, living arrangement, and employment. Clinical factors included primary psychiatric diagnosis, and service history with JMHSS (i.e., both total number of service episodes and length of stay). Referral pathway (i.e., step-up from home/community, or step-down from hospital) was the sole systemic factor available for analysis.

Second, for the primary aim, among those who had ‘paired’ data (i.e., both at entry and exit), change outcome measures were tested for statistical significance using paired t-test. Magnitude of change was also expressed as percentage of change relative to baseline for added context. Statistical predictivity of patients’ characteristics in change outcomes was assessed using generalized linear modelling, given the response variable being continuous. For the secondary aim, among patients who completed an Exit Questionnaire, their satisfaction with service was profiled using descriptive statistics and column charts. Simple Pearson’s correlation coefficients were also calculated for preliminary assessment of the associations between patients’ service satisfaction and their change outcomes. Where relevant, these associations were investigated further using generalized linear modelling.

As the outcome measures were available and relevant at the episode level, all the main analyses were conducted at the episode level accordingly. The only exception was the profiling/ characterization of the patient cohort (the descriptive analysis) which was carried out at the (unique) patient level; this was consistent with Neami National’s data management practice where the patient’s characteristics at the most recent service episode were allowed to override those recorded for their earlier episode(s), should they have incurred multiple episodes. Quantitative data analyses were conducted in SAS (Statistical Analysis Systems), version 9.3. Statistical significance was set at α = 0.05; hence 95% Confidence Intervals (CIs) were presented in conjunction with estimates where appropriate.

Qualitative data on what patients valued about their JMHSS stays – used interchangeably ‘valued outcomes’ henceforth – were analyzed thematically according to a semi-grounded theoretical process as illustrated by Braun and Clarke (2006). The qualitative analysis process involved firstly a collective familiarity with all the response statements to the question ‘what was valuable to your stay?’ at service exit. This informed the nature of the enquiry which was to understand the type of personally valued outcomes that patients experienced as a result of their stay at the JMHSS, and to understand how these outcomes were facilitated. Secondly, the response statements were broken into smaller response units. For example the response “I learnt a lot about myself and seem to have got my medications right, as well the staff have been so caring and helpful through this tough time” was broken into 4 smaller units as follows: 1) learnt about self, 2) medications working, 3) caring staff, and 4) helpful staff. Each response unit was deemed equivalent to a unique idea within a statement. Response units were then coded and grouped into themes according to either A) valued personal process outcomes (for example, ‘learnt about self’), or B) facilitators of valued outcomes (for example, ‘caring staff’).

### Ethics approval

This study was approved by The University of Western Australia’s Human Research Ethics Committee (RA/4/1/8805).

## Results

### Description of patient cohort

A total of 551 service episodes, corresponding to 382 unique patients, between 1 July 2014 and 30 June 2016 were included for analysis. Patients’ characteristics are described in Table 1. Mean age was 37.5 (range 18–65) years, with the majority of patients being female (61%). The majority of patients (82%) did not identify themselves as Aboriginal, and were living in a less disadvantaged area (78%). More than half of the patients were born in Australia, and nearly half of all patients were living with family at service entry and had no dependent children. Only 7% reported to be employed at service entry. More patients were referred from the ‘Step Up’ pathway (59%) than from ‘Step Down’ (37%). Patients with depression made up the largest subgroup (34%) in terms of primary diagnosis. Nearly three-quarters have had only 1 admission episode with Neami. Mean length of stay was approximately 3.5 weeks.

**Table 1:** Profile description of 382 patients accessing JMHSS service (total 551 episodes) between 1 July 2014 and 30 June 2016

Characteristics	Frequency	%
**Demographic factors**		
Age (in years) $	Mean 37.5	SD 12.3
Gender		
Male	148	38.7
Female	233	61.0
*Not known*	*1*	*0.3*
Indigenous status		
Yes	10	2.6
No	313	81.9
*Missing*	*59*	*15.4*
Social economic disadvantage		
More disadvantaged	81	21.2
Less disadvantaged	298	78.0
*Not known*	*3*	*0.8*
Country of birth		
Australia	214	56.0
United Kingdom / New Zealand	48	12.6
Other European countries	4	1.0
Other countries	10	2.6
*Missing*	*106*	*27.7*
Living arrangement		
Lives alone	53	13.9
Lives with family	180	47.1
Lives with others	36	9.4
*Not stated / Missing*	*113*	*29.6*
Has dependent child		
Yes, lives with consumer	38	9.9
Yes, lives elsewhere	43	11.3
No	190	49.7
Not stated / Missing	111	29.1
Employment		
Not in the labour force	243	63.6
Unemployed	6	1.6
Employed	27	7.1
*Not stated / Missing*	*106*	*27.7*
**Systemic factor**		
Referral type		
Step Up	224	58.6
Step Down	142	37.2
*Missing*	*16*	*4.2*
**Clinical factors**		
# episodes		
1	279	73.0
2	65	17.0
3	21	5.5
4 or more (up to 7)	17	4.5
Length of stay (in days) $ §	Mean 24.8	SD 9.1
(based on episode, not patient)		
Primary diagnosis		
Schizophrenia	51	13.4
Schizo-affective disorder	17	4.5
Bipolar disorder	51	13.4
Personality disorder	67	17.5
Depression	130	34.0
Anxiety	17	4.5
Post-natal depression	1	0.3
Eating disorder	2	0.5
Other psychiatric disorder	22	5.8
Not known/ Missing	24	6.3

Unless otherwise stated (see §), statistics are presented for unique patients and reflect the most recent episode if the patient has multiple episodes during the reporting period. ^§^ indicates data based on episodes, not unique patients

^$^ indicates continuous characteristics, hence mean and standard deviation are reported (instead of frequency and % being reported for categorical characteristics)

### Patients’ self-reported recovery outcomes, via changes in K-10, GSES, and WSAS scores

Significant improvements were seen in all three self-reported outcome measures at service exit, compared to at service entry (Table 2). The most pronounced improvement was in the patients’ psychological distress level, which was a 20% reduction compared to the baseline measure, with an associated strong Cohen’s d index of 0.7 in absolute value. Improvement in general self-efficacy was of comparable magnitude. Improvement in work and social adjustment was also strongly significant statistically, although of more moderate magnitude, as indicated by the Cohen’s d index.

**Table 2:** Summary of scores on: Kessler Psychological Distress Scale (K-10), General Self-Efficacy Scale (GSE), and Social and Work Adjustment Scale (WSAS). Data are based on episodes (see n below), not unique patients

	Entry			Exit			Exit-Entry Difference				
	n	mean	*SD*	n	mean	*SD*	n pairs	mean (95% CI)	*p*-value	% change	Cohen’s d
**K-10**	441	31.5	*9.0*	230	24.1	*9.5*	181	−6.5 (−8.0, −4.9)	<.0001	−20.7%	−0.70
**GSES**	390	24.8	*5.7*	213	28.2	*5.6*	158	3.5 (2.6, 4.4)	<.0001	14.1%	0.62
**WSAS**	444	23.7	*9.1*	229	19.3	*10.4*	183	−3.0 (−4.3, −1.7)	<.0001	−12.8%	−0.31

Note: K-10 and WSAS measure dysfunction, hence negative (−) score changes are desired; whereas GSES measures function, hence positive (+) score change is desired

% change relative to Entry, or baseline

*CI* Confidence Interval

### Predictors of patients’ self-reported recovery outcomes

Table 3 shows patients with higher K-10 score (i.e., higher psychological distress) at baseline improved more than those with lower distress level at baseline, by 0.6 (95% CI 0.5, 0.7) point in score change for every 1-point increment in K-10 at baseline. Older age, female gender, non-Indigenous status, and having a dependent child living with the patient are also statistical predictors of greater improvement (or reduction) in K-10 scores (Table 3). Compared to patients with psychosis, those with personality disorder or with depression also had their psychological distress level improved to a greater extent. When all the significant predictive factors mentioned above were assessed concurrently in the same statistical model, only baseline K-10 scores remained significant predictors of K-10 score changes.

**Table 3:** Factors associated with improvement in K-10 scores (reduction desired) and GSES scores (increase desired), from service entry to exit

Factor	Comparison	Reduction in K10 score		Increase in GSES score	
		Estimate (95% CI)	*p*-value	Estimate (95% CI)	*p*-value
Baseline score	1-point increment in score	−0.60 (− 0.74, − 0.46)	< 0.0001 ***	− 0.57 (− 0.70, − 0.43)	<.0001 ***
Age	1-year increment in age	−0.15 (− 0.27, − 0.04)	0.0117 *	0.06 (− 0.01, 0.13)	0.0922
Gender	Female v Male	− 3.46 (− 6.61, − 0.32)	0.031 *	1.86 (0.06, 3.67)	0.0431 *
Indigenous status	Yes v No	11.60 (3.02, 20.17)	0.0083 **	−2.27 (− 7.14, 2.60)	0.3587
SES disadvantage	Yes v No	3.17 (−0.62, 6.96)	0.1010	−0.44 (−2.66, 1.78)	0.6980
Country of Birth	Australia v Overseas	0.21 (− 4.36, 4.78)	0.9266	−0.45 (−3.01, 2.12)	0.7316
Living arrangement	With family v Alone	−2.76 (−7.51, 2.00)	0.2541	1.28 (−1.51, 4.07)	0.3659
	With others v Alone	−2.17 (−9.06, 4.72)	0.5337	0.51 (−3.46, 4.48)	0.7989
Has dependent child	Child not with consumer v Child with consumer	8.62 (2.40, 14.83)	0.0069 **	−3.15 (−6.65, 0.35)	0.0775
	No child v Child with consumer	9.57 (4.63, 14.52)	0.0002 ***	−4.53 (−7.24, −1.81)	0.0013 **
In labour force	Yes v No	5.15 (− 0.72, 11.02)	0.0850	−1.60 (−4.60, 1.41)	0.2951
Referral type	Step-up v Step-down	−2.12 (−5.33, 1.09)	0.1935	1.13 (−0.67, 2.94)	0.2166
Primary diagnosis group	Bipolar v Psychosis	−2.99 (−8.74, 2.77)	0.3067	2.75 (−0.41, 5.91)	0.0872
	Personality v Psychosis	−5.96 (−11.52, −0.41)	0.0356 *	3.62 (0.80, 6.45)	0.0124 *
	Depression v Psychosis	−5.24 (−10.02, −0.45)	0.0322 *	3.86 (1.39, 6.34)	0.0024 **
	Other v Psychosis	−1.82 (−7.76, 4.12)	0.5467	0.66 (−2.37, 3.69)	0.6683
# Episodes	Single v Multiple	3.25 (−0.37, 6.87)	0.0785	−2.42 (−4.95, 0.10)	0.0594
Length of stay	1-day increment in stay	0.14 (−0.02, 0.31)	0.0832	−0.02 (− 0.13, 0.09)	0.7670

Statistics shown are for episodes, not unique patients

*, **, and *** indicate statistical significance at *p* < 0.05, *p* < 0.01, and *p* < 0.001, respectively

For GSES scores, baseline measure was also a strong statistical predictor of change at service exit, in that patients with higher GSE score (i.e., higher efficacy) at baseline improved less than those with lower efficacy at baseline, by 0.6 (CI 0.4–0.7) point in score change for every 1-point increment in GSES at baseline (Table 3). Older age, female gender, and having a dependent child living in the same household also enhanced the recovery or improvement in GSES measure. Patients with personality disorder or depression improved more than those with psychosis (Table 3). When all of the significant predictive factors were assessed concurrently in the same statistical model, only baseline GSE scores and primary diagnosis grouping remained significant predictors of GSES score changes.

Of the 13 factors assessed, only the WSAS baseline scores were predictive of the WSAS score change at service exit. Specifically, patients with higher WSAS score (ie higher impairment) at baseline improved more than those with lower impairment at baseline, by 0.3 (CI 0.2–0.5) point in score change for every 1-point increment in WSAS at baseline. No other factor was significant in predicting WSAS score changes.

### Patients’ satisfaction with JMHSS service

Up to 251 episodes (or 45.6% of all 551 episodes) had data on patients’ satisfaction reported via the Exit Questionnaire, hence analyzed. Potential biases, or differences, were checked for Exit Questionnaire completers versus non-completers. Additional file 2 shows comparable profiles between the two groups, except that those who completed the Exit Questionnaire on average stayed 3 days longer than those who did not.

**Fig. 1:**
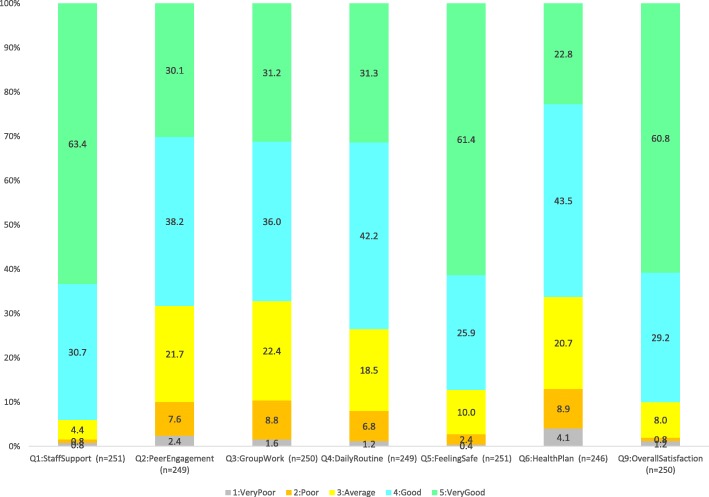
Distribution of patients’ ratings of satisfaction with service upon exit

As seen in Fig. 1, patients’ satisfaction with JMHSS service overall (Question 9) was rated highly, with 90% of the respondents reporting “Satisfied” or “Very Satisfied” with their stays. Similarly favourable ratings were also recorded for items on Staff support (94% positively rated) and Safety (87% positively rated). For the other four items (on Peer Engagement, Group Work, Daily Routine, and Health Plan), at least two-thirds of the respondents provided a positive rating.

All seven indexes of patients’ satisfaction with service positively and significantly correlated with the patients’ reduction in psychological distress (Table 4). Improvement in self-efficacy positively and significantly correlated with five of the seven indexes of service satisfaction (i.e., all but Questions 2 and 5, for peer engagement and feeling safe, respectively). Improved work and social adjustment (i.e., reduced impairment in work and social domains) only correlated significantly and positively with the patients’ rating of confidence with their health plan (Question 6).

**Table 4:** Correlations between patients’ Exit Questionnaire ratings (of satisfaction with service) and their score changes on K-10, GSES, and WSAS

		Change in K10 score	Change in GSES score	Change in WSAS score
**ExitQ1**	rho	−0.332	0.172	−0.006
**(Staff Support)**	*p*-value	<.0001 ***	0.0466 *	0.9375
	n	156	134	157
**ExitQ2**	rho	−0.231	0.147	−0.134
**(Peer Engagement)**	*p*-value	0.0037 **	0.0912	0.094
	n	156	134	157
**ExitQ3**	rho	−0.297	0.190	−0.009
**(Group Work)**	*p*-value	0.0002 ***	0.0284 *	0.9152
	n	155	133	156
**ExitQ4**	rho	−0.374	0.194	−0.024
**(Daily Routine)**	*p*-value	<.0001 ***	0.0261 *	0.7644
	n	154	132	155
**ExitQ5**	rho	−0.253	0.161	−0.099
**(Feeling Safe)**	*p*-value	0.0014 **	0.0624	0.2175
	N	156	134	157
**ExitQ6**	rho	−0.351	0.234	−0.305
**(Health Plan)**	*p*-value	<.0001 ***	0.0075 **	0.0001 ***
	n	153	130	153
**ExitQ9**	rho	−0.353	0.212	0.012
**(Overall Satisfaction)**	*p*-value	<.0001 ***	0.0145 *	0.8801
	n	155	133	156

Statistics shown are for episodes, not unique patients

*, **, and *** indicate statistical significance at *p* < 0.05, *p* < 0.01, and *p* < 0.001, respectively

Given that Question 6 taps on the core focus of Neami’s service model, and that it is most consistently associated with the three outcome measures (score changes), we assessed its effect further with generalized linear modelling. Patients who were most confident (rating 5) with their health plan on average observed a further reduction in psychological distress level (estimate 6.3 points; 95% CI 2.3, 10.3; *p* = 0.0022), relative to those who were less confident (ratings 1–4). Similarly, patients with highest confidence with their health plan also reported a greater reduction in work and social impairment by a further 5.3 points (95% CI 1.9, 8.6; *p* = 0.0021). For general self-efficacy, however, there was only a marginally significant effect of confidence with health plan (*p* = 0.0538).

### Patient exit interview qualitative feedback

There were 235 episodes (or 42.6% of all 551 episodes) contributing qualitative responses to the question ‘what was valuable to your stay?’ via the Exit Questionnaire. The 235 responses were broken into 531 smaller response units, which were subsequently categorized into two broad themes of (A): Valued outcomes, and (B): Outcome facilitators. As seen in Table 5, each broad theme encompasses four subthemes (Table 5). That is, four top valued outcomes from the patients’ stay at JMHSS were: (i) time and space to recuperate, (ii) perspective and insight; (iii) focus and direction; and (iv) changes, particularly in relation to establishing connections and supports, establishing a routine, gaining knowledge, self- validation and confidence. Facilitators of these valued outcomes included: (i) the environment dedicated to improving mental health, (ii) staff attributes, (iii) program activities, and (iv) interactions with others.

**Table 5:** Themes and ideas derived from participants’ responses for (A) what outcomes they valued from their stays at the Joondalup Mental Health Step-Up Step-Down (JMHSS) and (B) facilitators of those valued outcomes

Theme	Sub-Theme	Idea
A. Valued Outcomes (*n* = 177 response units)	Change	Connections and support
	(*n* = 84 response units)	Out of rut and into routine
		Knowledge
		Validation
		Strength and confidence
	Focus & Direction	Clarify what’s important/ values…
	(*n* = 37 response units)	New coping skills and strategies
		Make plans and set goals
	Perspective & Insight	Sort and gather thoughts
	(*n* = 30 response units)	Learned a lot of things about …
		Put things into perspective
	Time & Space to Recuperate	Time to myself
	(*n* = 26 response units)	Distance from … and Space to…
		Peace and relaxation
B. Facilitators of Valued Outcomes (*n* = 354 response units)	Program Activities	Optimal Health Program
	(*n* = 130 response units)	One-on-one time with staff
		Group time
		Relaxation/mindfulness techniques
		Goal setting and planning
		Cooking
		Independence
		Routine
		Activities and outings
	Staff Attributes	Supportive, helpful, caring & empathic
	(*n* = 92 response units)	Respect for choice and autonomy
		Approachable, able to talk to and be heard
	Interactions & Connections	Opportunity to be social
	(*n* = 69 response units)	Hearing and learning from peers
		Support and validation
		Friendships
	Environment	Safety
	(*n* = 63 response units)	Own space/ privacy when needed
		Comfortable and relaxing

## Discussion

### Summary and interpretation of key findings

This study provides evidence that receipt of service at the JMHSS was strongly associated with significant improvement in patients’ psychological wellbeing (i.e., reduced distress), general sense of self-efficacy, and work and social adjustment. There was a strong and persistent baseline effect present across the three measures (K10, GSE, and WSAS), which showed that the less well patients benefited more from the service. This makes sense, as the further one is from the ‘ideal’ position, the more ‘room’, or potential, one has to improve, or to get there. Older patients and female patients benefited more from the services, compared to their younger or male counterparts, respectively. Further analyses (data not shown) confirmed the underlying baseline effect, which stipulated that older or female patients had reported poorer health status (i.e., higher psychological distress and lower self-efficacy) at baseline than their respective younger or male peers. Parenting responsibility and role model expectation appeared to serve as a protective factor for the patient’s psychological wellbeing and general self-efficacy during their recovery journey with JMHSS. There was some mild indication that repeated patients (i.e., those with more than one service episode) might have benefited more, via slightly greater reduction in psychological distress scores and increase in self-efficacy scores, than their single-episode peers. If true, this would not be a surprising finding, as anecdotally in focus groups run by Neami, patients did share that they appreciated using the JMHSS as a way of ‘top-up’ for improving their mental health.

Patients’ satisfaction with the service, as reported via the Exit Questionnaire, in general was high, with higher satisfaction generally correlating with greater improvements in the three outcome measures (reduced psychological distress, enhanced self-efficacy, and reduced impairment in work and social activities). Features that patients valued most from their stays with JMHSS include being afforded with the time and space to recuperate, gain perspectives, focus, and create changes in their lives. Facilitating these valued features were: aspects of the environment (physical space, atmosphere and approach), staff attributes, program activities, and interactions with other residents and staff. Thematically and conceptually, the patients’ qualitative feedback concurs with the three quantitative outcome measures. For example, reduction in psychological distress (measured via K-10) would be expected to be ‘linked’ to the patients having the time and space to recuperate. Similarly, gaining insight, focus, direction, and the ability to create change (such as establishing new routines, new coping strategies, support network) should foster one’s sense of self-efficacy (measured by GSES) as well as enhance one’s work and social functioning (measured by WSAS). Notably, patients who completed the Exit Questionnaire on average stayed three days longer than those who did not complete the questionnaire. This may be because longer stays afforded more opportunities for patients to be contacted to complete the Exit Questionnaire, although longer stays were not associated with more beneficial change outcomes, as already seen in the Results.

### Contribution to the literature - comparison with other studies

Findings from this study build on recent Australian literature for the value of the SUSD setting to patients, or service users, in terms of a) change outcomes identified by routinely administered assessment tools and b) qualitative feedback obtained from consumers who have accessed this type of service [4, 5, 8, 9]. They also add to recent efforts to form evaluation practices from program logic modelling [3]. Specifically, the three validated measures K-10, GSES, and WSAS were used as theoretically they were thought to reflect the desired changes in the key domains of the mental health patient’s wellbeing, namely reduced psychological distress, improved self-efficacy and work and social functioning. Whilst these measures may allow for comparisons between mental health services, the use of qualitative feedback adds a nuanced understanding of the outcomes that are valued by the patients – how these are experienced, and which aspects of service that facilitate these valued outcomes. Having time and space to recuperate in an environment dedicated to mental health and wellbeing whilst also being enabled to develop skills and confidence to face and manage future challenges, appears to be the principal process underpinning the positive change outcomes identified in this study. From a program logic modelling perspective, the assessment of change outcomes undertaken in this study suggest that there is a reasonable alignment between the program activities and service approach and the measures utilised in this setting to assess outcomes.

The added qualitative component to assess the patient’s satisfaction with the service, particularly the valued aspects of their stay, has taken one step further in the direction towards ‘recovery-orientation’ [42], and away from a negative, deficit- [28] and disability- orientation [43]. Literature from the motivation and behavioral sciences demonstrate that a condition for behavior is what attention is drawn to [21]. Translated to the mental health services field, there is a case for shifting the focus of measurement tools towards that of desired gains [21, 26], an idea also supported by studies that have actively involved service users [29].

### Strengths

On many counts, this study has several strengths compared to previous research in the field. Specifically, our study has a much larger sample, in contrast to several previous studies employing an effective sample size of up to 40–50 participants (e.g., [3, 5, 9]). The more sizeable sample allows for multiple outcomes to be assessed concurrently and informatively. To our knowledge, this is the first study in the field that has systematically assessed the statistical predictivity of several patient variables in change outcomes.

Assessment of relationships between patients’ satisfaction with service and their outcome measures has added another level of insight not only into the patients’ reception and perception of the service, but also the potential areas that need focusing to help enhance their recovery. Further, nuanced qualitative feedback from patients have afforded the researchers, service providers, and stakeholders alike with an expanded understanding of what matters most to them along their ‘recovery’ or change process, which in turn will help develop outcome measures that are more meaningful to the patients, for whom the services are designed.

### Limitations

There are limitations to our study. First, this is a retrospective cohort study with only ‘cases’ and no ‘controls’ to act as an appropriate comparator. One therefore is not able to ascertain if the observed positive changes would have also occurred (e.g., by default) in non-SUSD patients. Second, a belated inclusion of an independent research collaborator precluded applications of stringent research principles, such as the absence of express, informed, and written consent from patients regarding the use of their data for research purposes, and more robust data collection methods, as noted next,

Third, Neami’s current system of data recording and storage allows for the patient’s last record and associated details to override all previous records, if the patient has had more than one service episode. This inadvertently removes an amount of potentially valuable longitudinal data, especially those on more ‘dynamic’ characteristics of the patient (such as employment status). This data removal also prohibits the potential examination of associations between these dynamic characteristics and the patient’s recovery outcomes at each episode.

Fourth, the outcome measure WSAS may have not been the most optimal in measuring the patient’s change in work and social adjustment. Although there was strong evidence for improvement in levels of functioning (or reduced impairment) in the patient’s work and social activities post-JMHSS, there was no significant predictor of this, other than its baseline level. Coupled with this is the observation (from Table 1) that the vast majority of the patient cohort was “Not in the labour force”, which together implies that an alternative instrument may more meaningfully measure a patient’s recovery. Given that employment and/or vocational activities are central to the mental wellbeing for most people, including those with severe mental illness (e.g., [44]), it is important that changes related to such outcomes are meaningfully tracked, to help enable a comprehensive evaluation of the SUSD model.

Finally, the qualitative analysis was limited by the material gathered by the routine assessment performed retrospectively, which might have introduced potential for bias (for example, if the person administering the Exit Questionnaire was a staff member that the patient had had contact with, or received support from, during their stay). Nevertheless, as alluded to earlier, the qualitative component has provided a supplementary first-person narrative about the value of the SUSD model to those who access it. Furthermore, the qualitative data come from the same population as the rest of the study, thus allowing the opportunity to compare these two sets of data, quantitative and qualitative.

## Conclusion

Overall, this study has showcased compelling, albeit preliminary, evidence for the need for more transitional/ community services like the JMHSS program, with a strong focus on both prevention and recovery. The program has shown very promising outcomes for patients, both in terms of their change measures and their satisfaction with several aspects of the service. Findings in this study build on the emerging evidence base for the value of the SUSD setting in terms of positive outcomes for consumers, and contribute knowledge that may advance program logic modelling and evaluation practices for mental health SUSD settings.

From a service viewpoint, the following aspects, among others, may require further attention. First, procedures and systems need to be in place such that the individual patient’s needs can be continually monitored and effectively met. For example, this may require a delicate balance in the provision of clinical and psychosocial care, depending on the patient’s journey or progress. Second, patient intake needs to be reasonably balanced such that SUSD services do not become overflow wards for acute cases, as anecdotally noted. In the current study of the JMHSS, this was not the case, with almost 60% of admission episodes coming from the ‘intervention/preventative’ Step-up stream (instead of Step-down). Third, services need to ensure equity in access and accessibility to patients of Indigenous and/or other disadvantaged ethnic backgrounds. In our study, 2.6% of the patients self-identified as Indigenous, which is approximately in line with the 2.1% estimate from the 2016 census for Greater Perth [45]. Nevertheless, nearly 28% of patients did not have information on country of birth recorded, with an additional 2.6% of patients who were born outside of Australia, New Zealand, the UK, and other European countries.

In terms of research enhancement, further conceptualizations and/or assessments of patient outcomes in alignment with the expectations of both the patients, the service providers and funders, particularly with the aid of more rigorous qualitative research methods, would be beneficial and recommended. Lastly, extensions of similar studies, with the inclusion of an appropriate control group, would help elucidate the robustness of the findings.

## Supplementary information

**Additional file 1.** Sub-acute consumer exit questionnaire

**Additional file 2. **Comparing baseline profiles of Exit Questionnaire completers (*n*=251) versus non-completers (*n*=300)

## Data Availability

In upholding the principles of research integrity and replicability, the authors are willing to provide detailed instructions of how the data analyses were conducted. The source dataset for this manuscript is not available publicly because it contains sensitive information on patients. Requests to access the dataset should be directed to the Data Custodian, Neami National, via their representatives: Keren Wolstencroft *keren.wolstencroft@neaminational.org.au* or Priscilla Ennals *priscilla.ennals@neaminational.org.au*.

## Abbreviations

SUSD: Step-Up Step-Down (model of care or service)
JMHSS: Neami Joondalup Mental Health Step-Up Step-Down Service
PARCS: Prevention and Recovery Services
K-10: Kessler Psychological Distress Scale (10-item version)
GSES: General Self-Efficacy Scale
WSAS: Work and Social Adjustment Scale
