# Supplementary material for: Step-up, step-down mental health care service: evidence from Western Australia’s first – a mixed-method cohort study
Source: BMC Psychiatry. 2020 May 11;20:214. doi: 10.1186/s12888-020-02609-w (PMC7216384; doi:10.1186/s12888-020-02609-w)
Supplement: Supplementary file 1 — Additional file 1. Sub-acute consumer exit questionnaire [file 12888_2020_2609_MOESM1_ESM.pdf]

## Sub-acute consumer exit questionnaire

| Name / UR number: | Date: |
|---|---|

1. How would you rate the level of support provided by the team during your stay?

| Very Good | Good | Average | Poor | Very Poor |
|---|---|---|---|---|

2. How would you rate your experience of engaging with other consumers during your stay?

| Very Good | Good | Average | Poor | Very Poor |
|---|---|---|---|---|

3. How would you rate your experience of being involved in group work with other consumers during your stay?

| Very Good | Good | Average | Poor | Very Poor |
|---|---|---|---|---|

4. How did you find the daily routine/structure during your stay?

| Very Good | Good | Average | Poor | Very Poor |
|---|---|---|---|---|

5. How safe did you feel here?

| Very Safe | Safe | Undecided | Unsafe | Very Unsafe |
|---|---|---|---|---|

6. How would you rate your level of confidence in now using your Health Plan to help you with keeping well?

| Very Confident | Confident | Unsure | Somewhat Confident | Not at all Confident |
|---|---|---|---|---|

| |
|---|
| 7. What was valuable about your stay? |
| 8. What might improve your experience of staying here? |
| 9. Overall how satisfied were you with your stay?<br><br>Very Satisfied · Satisfied · Neither Satisfied nor dissatisfied · Dissatisfied · Very Dissatisfied |
| 10. Would you like to make any other comments about your stay? |

Thank you for you valuable feedback. We sincerely appreciate your honest opinion and will take your input into consideration in providing services in the future.
