# Supplementary material for: Step-up, step-down mental health care service: evidence from Western Australia’s first – a mixed-method cohort study
Source: BMC Psychiatry. 2020 May 11;20:214. doi: 10.1186/s12888-020-02609-w (PMC7216384; doi:10.1186/s12888-020-02609-w)
Supplement: Supplementary file 2 — Additional file 2. Comparing baseline profiles of Exit Questionnaire completers (n=251) versus non-completers (n=300) [file 12888_2020_2609_MOESM2_ESM.docx]

Appendix II

Comparing baseline profiles of Exit Questionnaire completers (n=251) versus non-completers (n=300)

| **Characteristics** | **Completers** | | **Non-completers** | | | **p-value** |
| --- | --- | --- | --- | --- | --- | --- |
| **DEMOGRAPHIC** |  |  |  |  | |  |
| **Age ^$^** | Mean 37.6 | SD 12.6 | Mean 38.7 | | SD 11.7 | 0.3114 |
| **Gender** |  |  |  |  | | 0.1115 |
| Female | 152 | 60.6% | 202 | 67.3% | |  |
| Male | 98 | 39.0% | 98 | 32.7% | |  |
| Missing | 1 | 0.4% | 0 | 0.0% | |  |
| **Indigenous status** |  |  |  |  | | 0.4764 |
| Yes | 7 | 2.8% | 6 | 2.0% | |  |
| No | 203 | 80.9% | 260 | 86.7% | |  |
| Missing | 41 | 16.3% | 34 | 11.3% | |  |
| **Socio-economic disadvantage** |  |  |  |  | | 0.3086 |
| More disadvantaged | 53 | 21.1% | 52 | 17.3% | |  |
| Less disadvantaged | 196 | 78.1% | 240 | 80.0% | |  |
| Missing | 2 | 0.8% | 8 | 2.7% | |  |
| **Born in Australia** |  |  |  |  | | 0.8131 |
| Yes | 135 | 53.8% | 188 | 62.7% | |  |
| No | 41 | 16.3% | 54 | 18.0% | |  |
| Missing | 75 | 29.9% | 58 | 19.3% | |  |

Appendix II (Cont’d)

| **Characteristics** | **Completers** | | | **Non-completers** | | **p-value** | |
| --- | --- | --- | --- | --- | --- | --- | --- |
| **Living arrangement** |  |  |  | |  | | 0.2300 |
| Alone | 33 | 13.1% | 48 | | 16.0% | |  |
| With others | 22 | 8.8% | 43 | | 14.3% | |  |
| With family | 125 | 49.8% | 151 | | 50.3% | |  |
| Missing | 71 | 28.3% | 58 | | 19.3% | |  |
| **Dependent children** |  |  |  | |  | | 0.8153 |
| No | 125 | 49.8% | 175 | | 58.3% | |  |
| Yes, does not live with patient | 32 | 12.7% | 38 | | 12.7% | |  |
| Yes, lives with patient | 24 | 9.6% | 31 | | 10.3% | |  |
| Missing | 70 | 27.9% | 56 | | 18.7% | |  |
| **Employed** |  |  |  | |  | | 0.9323 |
| Yes | 22 | 8.8% | 29 | | 9.7% | |  |
| No | 165 | 65.7% | 212 | | 70.7% | |  |
| Missing | 64 | 25.5% | 59 | | 19.7% | |  |
| **SYSTEMIC** |  |  |  | |  | |  |
| **Referral type** |  |  |  | |  | | 0.1338 |
| Step up | 147 | 58.6% | 191 | | 63.7% | |  |
| Step down | 96 | 38.2% | 95 | | 31.7% | |  |
| Missing | 8 | 3.2% | 14 | | 4.7% | |  |

Appendix II (Cont’d)

| **Characteristics** | | **Completers** | | | **Non-completers** | | | **p-value** | |  |
| --- | --- | --- | --- | --- | --- | --- | --- | --- | --- | --- |
| **CLINICAL** |  | |  |  | |  | | |  |  |
| **Length of stay ^$^** | Mean 26.4 | | SD 9.1 | Mean 23.4 | | | SD 8.9 | | 0.0001 *** | |
| **Primary diagnosis** |  | |  |  | |  | | | 0.7347 |  |
| Psychosis | 41 | | 16.3% | 58 | | 19.3% | | |  |  |
| Bipolar | 37 | | 14.7% | 46 | | 15.3% | | |  |  |
| Personality | 45 | | 17.9% | 63 | | 21.0% | | |  |  |
| Depression | 88 | | 35.1% | 93 | | 31.0% | | |  |  |
| Other | 26 | | 10.4% | 30 | | 10.0% | | |  |  |
| Missing | 14 | | 5.6% | 10 | | 3.3% | | |  |  |
|  |  | |  |  | |  | | |  |  |

Statistics are shown for episodes, not unique patients.

^$^ indicates continuous measures, hence means and standard deviations are presented. For all other characteristics, which are categorical, counts and percentages are presented.

*** indicates statistical significance with p<0.001.
